# Arthritis and the role of endogenous glucocorticoids

**DOI:** 10.1038/s41413-020-00112-2

**Published:** 2020-09-08

**Authors:** Eugenie Macfarlane, Markus J. Seibel, Hong Zhou

**Affiliations:** Bone Research Program, ANZAC Research Institute, The University of Sydney, Camperdown, NSW Australia

**Keywords:** Diseases, Physiology

## Abstract

Rheumatoid arthritis and osteoarthritis, the most common forms of arthritis, are chronic, painful, and disabling conditions. Although both diseases differ in etiology, they manifest in progressive joint destruction characterized by pathological changes in the articular cartilage, bone, and synovium. While the potent anti-inflammatory properties of therapeutic (i.e., exogenous) glucocorticoids have been heavily researched and are widely used in clinical practice, the role of endogenous glucocorticoids in arthritis susceptibility and disease progression remains poorly understood. Current evidence from mouse models suggests that local endogenous glucocorticoid signaling is upregulated by the pro-inflammatory microenvironment in rheumatoid arthritis and by aging-related mechanisms in osteoarthritis. Furthermore, these models indicate that endogenous glucocorticoid signaling in macrophages, mast cells, and chondrocytes has anti-inflammatory effects, while signaling in fibroblast-like synoviocytes, myocytes, osteoblasts, and osteocytes has pro-inflammatory actions in rheumatoid arthritis. Conversely, in osteoarthritis, endogenous glucocorticoid signaling in both osteoblasts and chondrocytes has destructive actions. Together these studies provide insights into the role of endogenous glucocorticoids in the pathogenesis of both inflammatory and degenerative joint disease.

## Introduction

Arthritis is a collective term for over 100 joint diseases which are often chronic in nature and progressively debilitating if not managed and treated appropriately. While the exact global estimate of arthritis prevalence is difficult to discern due to the variety of diseases comprised under the term, it has been reported that rheumatoid arthritis (RA) and osteoarthritis (OA), the two most common forms, affect over 19 and 300 million people worldwide, respectively.^1^ Given the high incidence, degree of disability caused and the cost of treatment, arthritis accounts for a large proportion of health care expenditure worldwide.^2,3^

RA is a systemic autoimmune disease that can affect any joint, but mostly presents in a symmetrical manner in the hands, wrists, and feet. RA is primarily driven by a significant synovial inflammatory response involving the innate and adaptive immune system.^4^ OA, in contrast, is a predominantly degenerative disease, usually involving larger weight-bearing joints such as the knees and hips, as well as small joints of the hands and spine. Unlike RA, OA occurs in an asymmetrical pattern and aging is a major risk factor.^5^ OA is not primarily driven by an inflammatory reaction, and typically involves local pathological changes in the articular cartilage and subchondral bone of individual joints.

Glucocorticoids have been used as pharmacotherapeutics for RA and OA for more than 60 years, regardless of the fact that both diseases differ significantly in pathophysiology. This is possibly because the discovery of the potent anti-inflammatory effects of glucocorticoids was arguably one of the most important breakthroughs in the treatment of RA, and was historically perceived as a cure for “arthritis” by virtue of its effects on inflammation and restoring joint function.^6–8^ With their initial success, glucocorticoids have since been used to treat a variety of other inflammatory diseases. However, the recognition of severe adverse effects such as glucocorticoid-induced osteoporosis, metabolic disturbances (diabetes, atypical fat accrual, and dyslipidemia), skin atrophy, and hypertension has led to recommendations that glucocorticoids only be used at low doses and over short periods of time.^9,10^ Furthermore, the use of intra-articular glucocorticoid injections in OA remains controversial, with current evidence indicating injections have no therapeutic benefit and may actually worsen joint pathology in the long-term.^11,12^ This is a rather dismal situation for OA patients, who apart from analgesics, physiotherapy, and eventual joint replacement surgery have no disease-modifying treatment options available. This lack of effective therapeutics is primarily due to gaps in our understanding of the susceptibility and progression of arthritis, including the function of endogenous glucocorticoids. Major insights into the role of endogenous glucocorticoids in RA and OA have been derived from recent experiments utilizing genetically modified mouse models. These models suggest that different joint cell populations mediate arthritis via glucocorticoids in specific ways, which can either worsen or attenuate the disease.

Therapeutic glucocorticoids also have actions in other forms of arthritis including ankylosing spondylitis which affects the anterior ligament and facet joints of the spine;^13^ psoriatic arthritis, a chronic inflammatory joint disease associated with psoriasis;^14^ septic arthritis, which results from bacterial or viral infection of the joint;^15^ and gout, a metabolic condition caused by the build-up of uric acid in joint tissues.^16^ As there are limited studies to date that explore endogenous glucocorticoids in these less common forms of arthritis, this review will focus on the role of endogenous glucocorticoids in RA and OA. We will first compare the pathological features of these two diseases to highlight why glucocorticoid treatment has been broadly successful in RA, yet controversial in OA. We then review endogenous glucocorticoid mechanisms of action, their regulation and our current understanding of their role in each disease.

## Rheumatoid arthritis

RA is a chronic autoimmune disease, driven by pro-inflammatory changes in both the innate and adaptive immune system. Symptoms include synovitis, morning stiffness and pain, often involving the metacarpophalangeal and metatarsophalangeal joints in a symmetrical pattern, although any joint can be affected during the course of the disease.^17,18^ Early diagnosis and treatment with glucocorticoids, disease-modifying antirheumatic drugs (DMARDs) and nonsteroidal anti-inflammatory drugs (NSAIDs) can prevent or at least delay joint damage and functional decline.^17,19–22^

A hallmark feature of RA is the abnormal production of autoantibodies including rheumatoid factor (RF) and anti-citrullinated protein antibodies (ACPAs) which are detectable up to 10 years before symptoms of arthritis develop.^23^ This stage of the disease is termed “preclinical RA” as ACPA seropositivity does not always lead to joint disease.^24^ RF was the first autoantibody identified in patients with RA and targets the Fc portion of IgG. In contrast, ACPAs which are detectable in over two thirds of patients, are autoantibodies that recognize posttranslationally modified (citrullinated, homo-citrullinated, and acetylated) proteins and are associated with accelerated disease progression.^25^ Emerging evidence indicates that ACPAs are more than simply a biomarker and can also play a causal role in RA pathogenesis through interactions with immune and bone cells.^26–28^

The presence of these autoantibodies is caused by an interplay between genetic susceptibility in genes that code for human leukocyte antigen (HLA)^29^ and environmental factors such as smoking,^30,31^ periodontitis^32^ and certain gut microbes.^33,34^ More than 90% of RA patients carry at least one of the *HLA-DRβ1* alleles, with two alleles conferring higher risk of severe disease.^35–37^ However, these alleles are only associated with RA risk in ACPA-positive patients.^38,39^ A polymorphism in the *glucocorticoid receptor* gene has been found to be associated with RA and is thought to contribute to altered glucocorticoid regulation and sensitivity, yet further studies are required to determine if this polymorphism increases susceptibility to RA.^40^

### Pathological features of rheumatoid arthritis

**Fig. 1:**
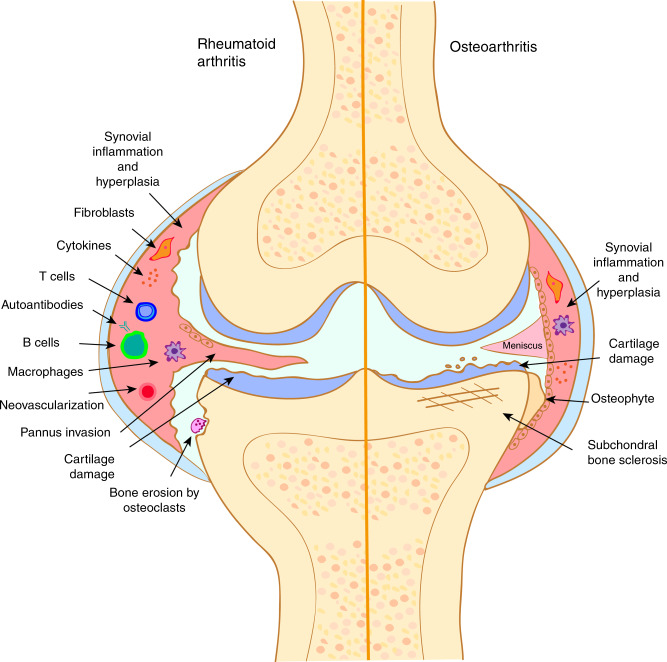
Comparison of the pathological features of rheumatoid arthritis and osteoarthritis. Rheumatoid arthritis is characterized by an inflammatory process mediated by innate, adaptive, and stromal autoimmune responses. The pannus, which is formed by activated synovial cells (“mesenchymal transformation”) invades and degrades the cartilage, while increased osteoclast activation results in bone erosion. In contrast, osteoarthritis is primarily a degenerative disease involving age-related changes in articular cartilage and bone structure and function that lead to subchondral bone sclerosis, ectopic bone (osteophyte) formation and cartilage destruction. Local synovial inflammation is a secondary feature seen in some cases of osteoarthritis

During preclinical RA, when symptoms of arthritis are not yet present, the synovium that encapsulates the joint remains unaffected.^41^ With the onset of joint swelling, stiffness and pain, the synovium becomes hypertrophic from the extensive proliferation of synovial fibroblast-like and macrophage-like cells and infiltration of innate and adaptive inflammatory cells including macrophages, T and B cells. This enlarged and thickened synovium is known as the “pannus” and can behave like an invasive tumor that expands into the joint space, attaches to the cartilage surface (cartilage–pannus junction) and invades and destroys the cartilage and underlying bone.^42–48^ In addition, hypoxia and increased cytokine and chemokine signaling cause the pannus to become highly vascularized, enabling continued migration of inflammatory lymphocytes, plasma cells and macrophages and subsequent progression of RA^49^ (Fig. 1).

Cells from both the innate and adaptive immune system and their respective cytokine profiles have been well studied and found to play pivotal roles in the initiation and perpetuation of inflammation and pannus formation. Fibroblast-like synoviocytes (FLS) are key mediators of pannus formation in their ability to transform and become highly mobile and invasive. FLS can grow in anchorage-free conditions^50^ and express adhesion molecules such as β1 integrins that interact with cartilage.^51^ Importantly, a study by Lefèvre et al.^52^ demonstrated that FLS from RA, but not OA patients can move between joints via the circulation, attach to healthy cartilage and cause its destruction.

Synovial macrophages are abundant and highly active innate immune mediators of synovitis, cartilage degradation, and bone loss in RA.^53^ Tumor necrosis factor α (TNF-α), and interleukins 1 and 6 (IL-1, IL-6), which are traditionally produced in higher quantities by M1 type pro-inflammatory macrophages, are elevated in RA.^54^ In addition, it has been shown that ACPAs are able to directly trigger the secretion of TNF in macrophages by binding to their Fc receptor (FcγR).^26^ A recent study by Fukui et al.^55^ also found that ACPA-positive RA patients had higher ratios of M1/M2 monocytes compared to ACPA negative-RA patients and this M1/M2 ratio positively correlated with IL-6 production and osteoclastogenesis. Thus, ACPAs may trigger distinct innate and adaptive inflammatory pathways that drive RA pathogenesis.

One of the main focuses of RA adaptive immunity has been the role of helper T-cell type 17 (T_H_17) which releases the pro-inflammatory cytokine IL-17.^56^ IL-17 is present in high levels in RA synovial fluid^57^ and its crucial role in the pathogenesis of RA has been demonstrated in studies where IL-17 deficient mice do not develop RA.^58^ Notably in 2017, Pfeifle et al.^59^ showed that IL-23 activated T_H_17 cells can also release IL-21 and 22 which interferes with B-cell antibody IgG sialylation and results in subsequent production of a type of pathogenic ACPA. This shift from nonpathogenic to pathogenic ACPAs is thought to be mediated by expression of the B-cell enzyme sialyltransferase (ST6GAL1), which results in inadequate glycosylation of ACPAs.^59^

The actions of these innate and adaptive immune cells together result in a highly inflammatory and catabolic joint microenvironment. As such, the destruction of cartilage in RA mostly occurs in areas adjacent to the hyperplastic synovial pannus, at the cartilage–pannus junction where synovial and immune cells release various cytokines and matrix-degrading enzymes^48,60–66^ (Fig. 1). One of the earliest changes evident in the cartilage matrix structure is the loss of proteoglycans and glycosaminoglycans which is thought to facilitate the attachment and invasion of the pannus.^67,68^ Chondrocytes, which play an essential role in replenishing matrix components and maintaining cartilage structural integrity, are targeted by several cytokines (TNF-α and IL-1) in the RA microenvironment. These cytokines stimulate chondrocytes to increase nitric oxide production^69^ and release pro-inflammatory signals (IL-6^70^ and matrix metalloproteinases MMPs^61^) which further enhance joint inflammation and perpetuate cartilage breakdown.^71^ Cartilage has limited regenerative potential and when the cartilage is degraded, chondrocytes undergo apoptosis resulting in inadequate matrix repair and irreversible damage.^72^ Interestingly, inhibition of chondrocyte activity in vitro has been shown to significantly decrease FLS invasiveness through an IL-1 mediated pathway,^71^ indicating that chondrocyte and synoviocyte interactions are integral in the prevention of cartilage damage and can modulate RA disease progression.

The loss of periarticular bone is also a consequence of the pro-inflammatory joint microenvironment. Bone erosion which can be detected early after the onset of joint inflammation and swelling,^73^ affects ~75% of RA patients within the first 2 years of diagnosis^74^ and contributes greatly to the loss of joint function and disability.^75^ Bone erosions are typically located adjacent to the cartilage–pannus junction and are commonly found in regions prone to mechanical stress such as the second and third metacarpals.^76,77^ Local inflammation caused by secretion of various cytokines, promotes monocyte migration into the joint, and osteoclast differentiation for bone resorption, resulting in an abundance of osteoclasts at the cartilage–pannus junction^78,79^ (Fig. 1). Nuclear factor kappa-B (NF-κB) (RANK) ligand (RANKL) expression is localized to areas of bone erosion where osteoprotegerin (OPG, osteoclastogenesis antagonist) expression is limited, suggesting a local microenvironment that favors osteoclast activation, but limited bone formation at disease relevant sites.^80–82^ Interestingly, a second pathway in which osteoclasts may be activated involves interactions with ACPAs in the joint. This is thought to occur because osteoclasts uniquely express protein arginine deiminases and citrullinated proteins under normal physiological conditions and ACPAs can bind to antigens such as citrullinated vimentin expressed on the surface of osteoclasts and promote osteoclastogenesis.^27,28^ This also helps explain the observation that bone loss can occur in the preclinical RA stage before signs of inflammation develop^83^ and may provide a link as to how systemic autoantibody production results in local joint pathology.

### Glucocorticoid treatment in rheumatoid arthritis

The discovery of the therapeutic benefit of glucocorticoids (GCs) as potent anti-inflammatories in 1949, revolutionized modern treatment regimens of inflammatory diseases including RA.^7^ However, the prolonged use of GCs, even at lower doses, is limited due to adverse effects such as osteoporosis, metabolic disturbances, skin atrophy, and hypertension.^84–86^ Despite this, GCs are still widely used today and remain an area of contention when assessing their benefit-risk profile. As such, the current recommendation for RA treatment is ≤7.5 mg·d^–1^ of prednisolone or equivalent for less than 10 weeks, to be used in combination with DMARDs.^22,87,88^

Notably, GC treatment has been shown to decrease the expression of synovial citrullinated proteins^89^ and stimulate the release of macrophage inhibitory factor^90^ to subsequently reduce synovial inflammation. In addition, GCs have a plethora of actions in immune regulation of T and B cells (reviewed in^91^). More recently, gene expression analysis has revealed that the effects of GC treatment are highly diverse and cell specific, with each cell having its own transcriptional profile.^92,93^ For example, Galon et al.^93^ found that GCs can upregulate or downregulate genes depending on the whether the cell was activated by a T-cell receptor. This not only highlights the complexity of pharmacological GC mechanisms of action, but also implies that the therapeutic and adverse effects of GCs may be mediated by certain cell subsets. In addition, while dexamethasone treatment can promote the survival of M2 type anti-inflammatory macrophages^94^ and suppress the production of various pro-inflammatory factors, treatment can also increase intracellular reactive oxygen species and MMP-13 expression in chondrocytes which leads to their apoptosis and cartilage matrix catabolism.^95^ Thus, it is plausible that the effects of endogenous GC signaling are also highly individualized depending on the activated cell type, and this has been demonstrated across various genetically modified mouse models that disrupt GC signaling in specific cell lineages.

## Osteoarthritis

Unlike RA, OA is a local degenerative disease, characterized by progressive changes in one or multiple diarthrodial joints. Disease progression is slow and manifests over several years before symptoms may develop. Most common symptoms include joint pain, stiffness, limited movement and loss of function, with 10% of patients unable to undertake daily life activities.^5,96,97^ Furthermore, joint pain and the severity of radiographical changes may not always correlate, meaning that once a diagnosis is made, joint damage may already be substantial and cannot be reversed by current treatments.^98^ To date, there are no therapeutics available that effectively prevent, halt, or even reverse OA. Current treatment strategies are mainly focused around pain relief via NSAIDs and intra-articular glucocorticoid injections. However, as OA is primarily a non-inflammatory disease, these anti-inflammatory therapies are usually not effective in the long-term. In advanced and debilitating cases, joint replacement surgery remains the only viable option.^99,100^ A major reason for the lack of effective prevention and medical treatment of OA is the limited understanding of the molecular and cellular mechanisms underlying the pathogenesis, perpetuation, and progression of the condition.

**Fig. 2:**
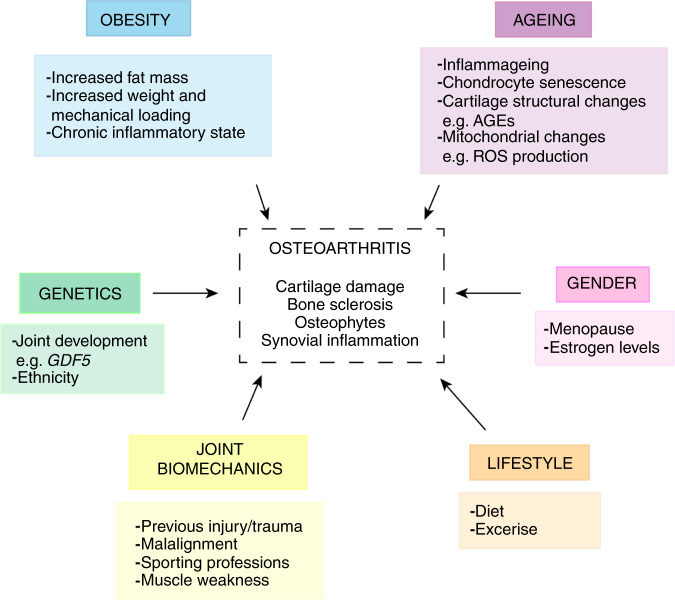
Osteoarthritis risk factors. AGEs advanced glycation end-products. ROS reactive oxygen species. *GFD5 growth/differentiation factor 5*

While RA can occur at any age, the incidence of OA increases rapidly after the age of 65^101^ (Table 1). It is thought that aging creates a vulnerable environment in the joint, that involves cellular senescence,^102–107^ mitochondrial dysfunction^108–115^ and alterations in the organization of the articular cartilage matrix,^116–120^ that together result in an accumulation of damage, in which there is a decline in the ability of cells to cope with abnormal stress.^121,122^ In combination with other circumstances such as biomechanical alterations in obesity or injury, joint repair may become insufficient, enabling the progression of OA^123–125^ (Fig. 2). The association between obesity and OA is widely acknowledged,^126–129^ with obese patients having greater than threefold risk of knee OA^130^ and over 40% at risk of requiring replacement surgery.^131^ For decades, this has been explained by the logical rationale that increased body weight, increases strain on joint loading and therefore acts as a mechanical contributor to the pathophysiology of OA.^132,133^ However, multiple studies have reported obesity-associated OA in non-weight-bearing joints such as the hand, suggesting the role of obesity-related chronic inflammation contributing to OA pathogenesis in the obese population.^130,134–137^ In addition, *the fat mass and obesity-associated* (*FTO*) gene has been shown to play a role in OA risk.^138^ However, in contrast to RA, where genetic variation in immune-related genes is causally associated with the disease, the role of genetics in OA is not as well defined. It is thought that genetic risk in OA is rather driven by a complex interaction between multiple small risk loci and the environment.^139–149^ As such, it is currently agreed that OA pathogenesis is highly heterogenous due to the combination of these risks, multiple possible initiating events, and the complexity of OA pathology.^150^

**Table 1:** Comparison between rheumatoid arthritis and osteoarthritis disease characteristics

Characteristics	Rheumatoid arthritis	Osteoarthritis
Major risk factors	Genetics *(MHC HLA alleles)* and environment (periodontitis, smoking, and gut microbes)	Aging, obesity, female gender, joint biomechanics, injury, and genetics *(GDF5)*
Age at onset	May occur at any age	Around 65 years +
Symptoms	• Painful, swollen, and stiff joints • Multiple joints usually in hands, wrists, elbows, and feet, with symmetrical presentation (affects both sides of the body) • Systemic reaction (fatigue and malaise)	• Tender, aching joints with some swelling/pain • Usually present on one side of the body in large weight-bearing joints such as the knee, hips, and spine • No systemic symptoms
Disease onset	Fast (weeks—months)	Slow (years)
Mechanism	Systemic autoimmune reaction (production of autoantibodies e.g., RF and ACPAs)	Local tissue degeneration due to aging, biomechanical, metabolic, and inflammatory changes
Pathological features	• Major synovial reaction (inflammation, hyperplasia, and pannus formation) • Destruction of cartilage by pannus invasion • Bone erosion (osteoclast activation)	• Cartilage damage (“wear and tear”) • Bone sclerosis • Bone marrow lesions (early stage) • Osteophytes • Synovial inflammation
Treatment	Glucocorticoids, NSAIDs and DMARDs (synthetic e.g., methotrexate and biological e.g., anti-TNF-α)	Pain management (NSAIDs, intra-articular glucocorticoid injections), lifestyle modification (exercise and diet), and joint replacement surgery

*MHC* major histocompatibility complex, *HLA* human leukocyte antigen, *GDF5* growth/differentiation factor 5, *RF* rheumatoid factor, *ACPAs* antibodies to citrullinated protein antigens, *NSAIDs* nonsteroidal anti-inflammatory drugs, *DMARDs* disease-modifying antirheumatic drugs, *TNF* tumor necrosis factor

### Pathological features of osteoarthritis

There are four hallmark features of OA: articular cartilage damage, subchondral bone sclerosis (increased bone volume but decreased bone mineralization), the formation of osteophytes (calcified osteochondral outgrowths), and secondary synovial inflammation. Changes in the cartilage and underlying bone are thought to play a causal role in the disease,^151–153^ while studies suggest that osteophytes only serve to stabilize the joint and are often associated with regions of misalignment, rather than playing an active role in disease progression.^154–160^

The most studied and well-known feature of OA is the progressive loss of the articular cartilage. Initially, the surface becomes irregular and roughened with visible fibrillations.^161,162^ This coincides with decreased histological staining of proteoglycans and increased level of water content, which leads to increased permeability, loss of the matrix integrity and weakening of the cartilage that may lead to further damage.^161,163^ In response to the tissue damage, chondrocytes undergo a phenotypic shift (characterized by cluster formation, hypertrophy, and calcification) and attempt to repair the damage by increased production of both matrix proteins and matrix-degrading enzymes.^161,164–166^ This repair response may last for years and in some patients may even slow the progression of the disease. In advanced and severe OA, deep fissures may extend to and expose the underlying calcified cartilage and bone.^161–163^ By this stage, the lack of protection from the degraded matrix combined with increased nitric oxide production causes chondrocyte apoptosis.^161,163,167^ In contrast to RA, where cartilage degradation is a direct result of pannus invasion and inflammation, the cause of cartilage destruction in OA is still an area of contention. Traditionally it was thought that OA was simply a degenerative disease caused by the continuous mechanical “wear and tear” of the articular cartilage, with the underlying hypothesis that advancing age causes an accumulation of microtrauma to the cartilage and chondrocytes.^163,168,169^ However, advances in diagnostic imaging and molecular biology have steadily shifted this paradigm suggesting that OA is a complex disease process involving multiple joint tissues, which ultimately leads to joint failure from overwhelming trauma and maladaptive repair.^150,170^

Current evidence suggests that early alterations in subchondral bone are a driving force of articular cartilage degradation.^152,168,171^ Studies in the 1970s and 80s initially identified that cartilage fibrillation did not always lead to OA and reported that stress could be induced in cartilage by local experimental alterations in subchondral bone.^168,172^ The advent of magnetic resonance imaging (MRI) further enabled the identification of bone marrow lesions (bone marrow edema),^173^ which seem to occur underneath severely degraded cartilage^152,174^ and strongly associate with progressive cartilage loss.^175–177^ Increasing evidence supports the use of bone marrow lesions as a powerful predictor of OA progression, especially as they can develop earlier than cartilage damage and before pain is present.^175,176,178^ Studies that disrupt important coordinators of bone remodeling such as transforming growth factor β (TGF-β), suggest OA involves a failure of an abundant osteoid matrix to mineralize normally.^153,179,180^ In early stages of OA during osteoclast resorption of bone, TGF-β is abnormally elevated in bone cells and attracts mesenchymal stem cells to migrate and form new bone at the site of resorption.^157,181,182^ Inhibition of TGF-β activity in bone can reduce aberrant bone formation and attenuate articular cartilage damage in an anterior cruciate ligament transection OA mouse model, further providing evidence that changes in the underlying bone drive cartilage damage.^179,180^ More recently, studies utilizing high-resolution peripheral quantitative computed tomography have identified microstructural changes in the subchondral bone of OA patients, also implicating abnormal bone remodeling as part of OA pathogenesis.^183,184^ In particular, Chen et al.^183^ found there was a reduction in rod-like trabeculae and thickening of plate-like trabeculae in human and guinea pig OA knees, which preceded cartilage damage in the guinea pig OA model.

Together these studies challenge the traditional view of OA as simply a degenerative cartilage disease, and suggest that OA also involves complex interactions between the underlying subchondral bone, which may occur prior to cartilage damage. Furthermore, cartilage damage and bone changes significantly differ between OA and RA. While joint destruction in RA is mediated by the adaptative and innate immune response, resulting in significant bone and cartilage loss, cartilage damage in OA is driven by a variety of age and biomechanical-related mechanisms particularly in the bone. Although inflammation in OA is less severe and fundamentally different than in RA, several studies have shown a positive association between synovial inflammation, cartilage loss and progression of OA.^182,185–187^ This is partly due to advances in MRI and ultrasound that have confirmed the role of synovitis in OA over the past few decades. Whether synovial inflammation is causative, or a consequence of joint failure remains unclear. However emerging evidence suggests that innate immune activation is an important driver of synovial inflammation and contributes to OA progression in early stages of the disease.^188^

### Intra-articular glucocorticoid injections as a treatment for osteoarthritis

Although the therapeutic benefit of GCs is well established in RA, their use in OA remains controversial.^189^ For example, two similarly designed random control trials that investigated the therapeutic effect of intra-articular steroid injections for knee OA yielded conflicting results. An earlier study by Raynauld et al.^190^ reported no detrimental effects of 3-monthly intra-articular injections of triamcinolone acetonide 40 mg on knee structure (measured radiographically), with injections significantly improving range of motion, but only slightly reducing pain compared to baseline and saline controls. Using a similar protocol, a later study by McAlindon et al.^11^ found injections had no effect on knee pain or function, and caused significantly greater cartilage volume loss (measured by MRI) compared to a saline placebo. The results of these two studies highlight the importance of selecting both the appropriate patient cohort and tool for measuring synovitis. While Raynauld et al.^190^ included patients solely based on radiographic changes with inflammation status unclear, McAlidon et al.^11^ only included patients with ultrasonographic evidence of joint effusion. Incongruent results like these may also be due to the fact that OA is primarily a non-inflammatory condition. In addition, there is emerging evidence that OA is a syndrome of different disease subtypes which differ in their pathogenesis, progression, and disease severity.^150,191,192^ Although studies have attempted to distinguish OA subtypes, each approach the problem in different ways based on either risk factors, symptoms, imaging or hypothesized pathomechanisms.^150,170,191–194^ This highlights the need for consensus among groups, which would aid in both communication across the field and advancements in more specific and effective treatment options.^150,195^ Thus, in future, rather than applying the same treatment to all OA patients, identifying a subset of patients with synovial inflammation through MRI may assist in understanding the efficiency of anti-inflammatory therapy such as intra-articular glucocorticoid injections or NSAIDs.

## Endogenous glucocorticoids

While the actions of exogenous GCs have been of major interest in arthritis research, the role of endogenous GCs has only gained attention in the past decade, owed at least in part to the development of genetically modified mouse models of disease. In particular, models that allow modification or disruption of endogenous GC signaling at the tissue level have provided further insight into how endogenous GCs mediate joint cell homeostasis during development, as well as in RA and OA pathology.

**Fig. 3:**
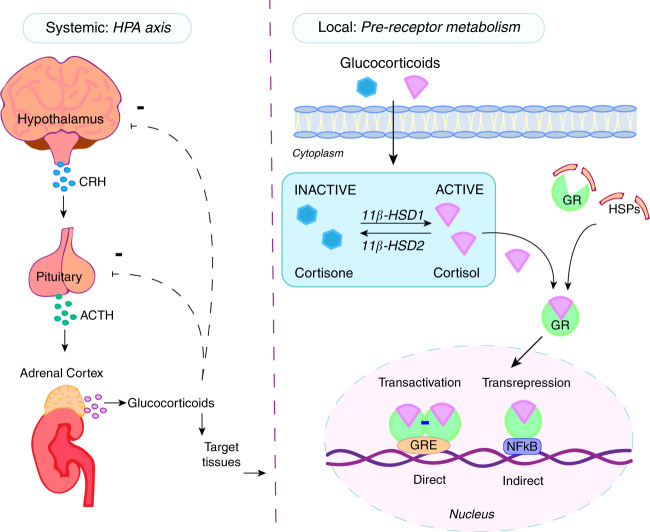
Systemic and local regulation of glucocorticoids (GCs). GCs are regulated both systemically through the hypothalamic–pituitary–adrenal (HPA) axis (left) and locally by intracellular enzyme metabolism (right). In the HPA axis, corticotropin-releasing hormone (CRH) is released from the hypothalamus, stimulating the secretion of adrenocorticotropic hormone (ACTH) from the anterior pituitary gland. ACTH binds to receptors in the adrenal cortex located above the kidney and stimulates GC synthesis and release into the bloodstream. Locally, GCs are metabolized into their active (triangle) or inactive (hexagon) form by two enzymes: 11β-hydroxysteroid dehydrogenase type 1 (11β-HSD1) and 11β-hydroxysteroid dehydrogenase type 2 (11β-HSD2) respectively. Active GCs bind to the glucocorticoid receptor (GR) complex with heat shock proteins (HSP) and other proteins. Upon binding, the GR undergoes a conformational change that allows its translocation into the nucleus. In the nucleus, the ligand-GR complex may bind to the DNA directly (via glucocorticoid response elements, GRE) or indirectly (via transcription factors such as nuclear factor kappa-B, NF-κB) to initiate transactivation or transrepression pathways

Endogenous GCs are steroid hormones physiologically secreted by the adrenals under the regulatory control of the hypothalamus and the pituitary gland (the “HPA axis”) (Fig. 3). They are essential for somatic development,^196–198^ maintenance of glucose metabolism,^199^ cognitive function,^200^ immunity,^201^ and skeletal homeostasis.^202,203^ As such, endogenous GCs play a pivotal role in whole body homeostasis and the physiological stress response.

The concentrations of circulating endogenous GCs follow a diurnal rhythm, with secretion highest upon wakening that steadily declines over the day under normal basal conditions. In response to changes in circadian rhythm, metabolism, and stress signals including systemic inflammation, the adrenal glands are stimulated via the hypothalamic–pituitary–adrenal (HPA) axis to produce and secrete GCs (Fig. 3). The HPA axis regulates the release of adrenal GCs through the paraventricular nucleus of the hypothalamus, which receives feedback from a variety of stimuli and controls the release of corticotropin-releasing hormone (CRH) and vasopressin. The release of these hormones stimulates the secretion of adrenocorticotropic hormone (ACTH) from the anterior pituitary gland, which binds to receptors on the adrenal cortex, resulting in the synthesis and release of GCs into the bloodstream. The HPA axis is maintained through negative feedback loops to reduce GC production when levels are high, preventing adverse effects from prolonged GC exposure.

While circulating GC levels are regulated by the HPA axis, intracellular concentrations are determined by the actions of the 11β-hydroxysteroid dehydrogenase enzymes (11β-HSDs). There are two distinct isozymes of 11β-HSD: type 1 (11β-HSD1) and type 2 (11β-HSD2), both of which metabolize GCs intracellularly prior to receptor binding^204^ (Fig. 3). 11β-HSD1 works predominantly as a reductase in vivo, and converts inactive cortisone (in humans) and 11-dehydrocorticosterone (in rodents) into active cortisol and corticosterone, respectively. This causes an intracellular increase in the level of *activated* GCs which can bind to the GR and consequently, amplify local GC signaling.^205^ 11β-HSD1 is widely expressed in tissues where GR density is relatively high, such as the liver, adipose and gonads,^205,206^ and is regulated by signaling changes in the local environment such as inflammation or aging, independent of systemic GC concentrations. In contrast, 11β-HSD2 works exclusively as a unidirectional dehydrogenase and catalyzes a reaction opposite to that of 11β-HSD1, by converting active GCs to their inactive metabolites. The expression of 11β-HSD2 is mostly restricted to mineralocorticoid tissues such as the kidney where it prevents GCs from binding to the mineralocorticoid receptor (MR) to ensure preferential aldosterone-MR binding.^207^

### Glucocorticoid receptor

GC actions in target tissues are determined by their plasma concentrations, pre-receptor metabolism via the action of the 11β-HSD enzymes, and GC interactions with the glucocorticoid receptor (GR). Given their lipophilic structure, GCs such as cortisol (in humans) and corticosterone (in rodents) can freely diffuse into the cell and bind to the cytoplasmic GR, resulting in a series of conformational changes that allow translocation of the ligand–GR complex into the nucleus to regulate gene expression.^208^ In the nucleus, the ligand-bound GR can either act directly or indirectly on DNA to activate or repress target gene expression^209,210^ (Fig. 3). When the GR acts directly with DNA, it forms a homodimer and binds to glucocorticoid response elements (GREs) within the promoter region of target genes to initiate transcription. In contrast, the monomeric GR binds, independently of DNA contact, to transcription factors such as NF-κB or activator protein 1 (AP-1) to regulate transcription. Since NF-κB and AP-1 are transcription factors activated by pro-inflammatory cytokines, it was initially proposed that this pathway was responsible for the anti-inflammatory effects of GC therapy, so called “transrepression”. Conversely, binding of the GR homodimer to GREs was believed to be associated with adverse metabolic effects and termed the “transactivation” pathway. However, this hypothesis is now recognized as oversimplistic and has been challenged by various studies utilizing dimerization deficient GR^dim^ mice with a single point mutation that impairs GR dimerization.^211^ These studies have found GR^dim^ mice are highly susceptible to sepsis characterized by increased pro-inflammatory cytokine expression and are resistant to GC treatment in an antigen-induced arthritis model of RA.^212–215^ Thus, these studies demonstrate that GR dimerization has essential anti-inflammatory effects in both septic shock and arthritis.

### Endogenous glucocorticoid actions in rheumatoid arthritis

Our current understanding of the actions of endogenous GCs in RA has come from clinical studies, in vitro studies using well-defined RA patient samples, and mostly in vivo animal models of RA. The induction of autoimmune arthritis in rodents usually involves the generation of an antibody-mediated immune response against specific antigens, and the activation of pro-inflammatory cytokines. In the case of collagen-induced arthritis (CIA), the antigen is cartilaginous type II collagen, which stimulates a substantial inflammatory response involving IL-17, TNF-α, and IL-1β.^216^ In the K/BxN serum-transfer arthritis (K/BxN STA) model, antibodies to glucose-6-phosphate isomerase are generated in a susceptible host and then transferred via serum injections to experimental mice. This generates inflammation, and again, TNF-α and IL-1β are essential for the development of arthritis.^217^ Notably, both these cytokines stimulate adrenal GC output^218^ and upregulate 11β-HSD1 activity in synovial fibroblasts and osteoblasts^219,220^ making these models valuable in understanding the role of endogenous GCs in RA. Currently, a variety of animal models have been used to mimic certain aspects of the adaptive and innate immune response to study endogenous GC actions in RA. For example, CIA and antigen-induced arthritis (AIA) are T-cell dependent models that involve the contribution of both adaptive and innate immune cells to initiate joint destruction. Conversely, in the human-TNF-transgenic (TNF-tg) mouse model, mice express high levels of human TNF-α and develop spontaneous and chronic arthritis.^216^ The collagen-antibody-induced arthritis (CAIA) and K/BxN STA mouse models also develop arthritis independently of T-cell activation and are therefore used to investigate how GCs mediate innate immune responses in joint destruction.

#### The HPA axis is dysregulated in patients with rheumatoid arthritis

Studies in the 1980s and early 90s initially found that the circadian rhythm of endogenous GC secretion was lost in RA patients with high disease activity, as reflected by markedly reduced cortisol levels throughout the day.^221^ This concept was in keeping with the later finding that RA patients exhibited a reduced serum cortisol response following CRH stimulation.^222^ These results indicated that the pathogenesis of RA involved a failure of the HPA axis to increase systemic GC concentrations in response to chronic inflammation.^222^ However, other studies have demonstrated that RA is associated with increased GC activation both systemically and locally, due to high 11β-HSD1 activity.^223,224^ Hardy et al.^223^ found that RA patients exhibited high 11β-HSD1 activity in synovial tissue and urine which was positively associated with the degree of systemic inflammation.

To further elucidate the role of systemic and local endogenous GCs in RA, experimental models of acute (K/BxN STA) and chronic (spontaneous K/BxN) arthritis were used in a recent study. In both models, mice exhibited a significant increase in pituitary 11β-HSD1 expression,^225^ leading to the hypothesis that an increase in local (pituitary) GC activity enhances negative feedback in the pituitary, which in turn might explain the abnormalities seen in the HPA axis of RA patients.^225^ The same study also found that chronic, but not acute arthritis, was associated with a reduction in hypothalamic CRH and vasopressin expression, further suggesting that chronic autoimmune arthritis is associated with dysfunctional HPA axis activity. However, as there was no difference in serum ACTH and GC levels in arthritic and non-arthritic mice,^225^ the dysregulation of the HPA axis may not reduce circulating GCs in this model. This was also confirmed by Hardy et al.^226^ who found that global deletion of 11β-HSD1 in TNF-tg mice (TNF-tg^11βKO^) did not alter circulating levels of GCs in chronic arthritis.

Taken together these studies demonstrate the complexity of the interaction between the HPA axis and circulating and local GCs in arthritis. In fact, local GC levels, which are determined by tissue-specific GC metabolism, often do not correlate with systemic GC concentrations. While systemic GC levels are either reduced or unchanged in RA, it appears that local GC activity in the inflamed joint is elevated.

#### Local inflammation upregulates 11β-HSD1 expression

The effects of endogenous GCs at the tissue and cellular level in RA have only drawn attention in the past decade. It is currently understood that pro-inflammatory cytokines including TNF-α, IL-1, and IL-6 which are elevated in the RA joint,^54^ increase local 11β-HSD1 expression and thereby, availability of active GCs. This regulation has been demonstrated in a clinical study, where anti-TNF-α therapy in RA patients decreased systemic 11β-HSD1 activity and also increased the activity of a glucocorticoid inactivating enzyme 5α-reductase.^224^ In a rat model of RA, synoviocyte 11β-HSD1 mRNA expression was more than 20-fold higher, and 11β-HSD1 displayed enhanced activity in activating corticosterone.^227^ In addition, treatment with TNF-α and IL-1β antagonists decreased synovial 11β-HSD1 mRNA upregulation and improved disease activity in these rats.^227^ This has been confirmed in other studies demonstrating that treatment with TNF-α or IL-1β can significantly increase 11β-HSD1 expression and activity in various cells including human osteoblasts,^220^ adipose stromal cells,^228^ and RA patient-derived synovial fibroblasts^219^ in vitro. Together these studies demonstrate that in arthritis, local 11β-HSD1 expression and activity is under the regulatory control of the pro-inflammatory microenvironment, driven by TNF-α and IL-1β. Importantly, this inflammation-induced upregulation of local GC signaling in the cell has the capacity to influence disease progression and severity.

#### Global inhibition of endogenous glucocorticoid signaling has predominately anti-inflammatory effects

Global inactivation of the *GR* gene leads to high perinatal lethality in knockout mice. Therefore, the contribution of global endogenous GC signaling to the susceptibly and severity of arthritis has been investigated through the use of pharmacological GR inhibitors, 11β-HSD1 knockout and GR dimerization (GR^dim^) mouse models. Treatment of Lewis rats with the GR inhibitor RU38486 results in higher susceptibility to streptococcal-induced arthritis, a model that mimics certain aspects of RA.^229^ Similarly, in Lewis rats with adjuvant-induced arthritis, global inhibition of 11β-HSD1 using carbenoxolone results in significant edema and upregulation of synovial inflammatory cytokines including TNF-α without changing plasma corticosterone levels.^227^ Together, these two studies suggest endogenous GC signaling may have local anti-inflammatory effects in RA. This is also supported by results from studies that utilize global 11β-HSD1 knockout mice. Using a K/BxN STA model, Coutinho et al.^230^ found that global 11β-HSD1 deletion resulted in earlier onset and slower resolution of arthritis, characterized by greater swelling, exostosis and inflammatory ganglion cysts when compared to wild-type mice. In the same study, the anti-inflammatory effects of 11β-HSD1 were also demonstrated in a thioglycolate-induced sterile peritonitis model of inflammation, where 11β-HSD1 knockout mice exhibited greater peritoneal inflammatory leukocyte infiltrates and more severe pleural pathology. Recently, Hardy et al.^226^ demonstrated that global deletion of 11β-HSD1 in a TNF-tg mouse model (TNF-tg^11βKO^) exacerbated joint swelling, pannus invasion, cartilage destruction, and systemic bone loss. Further analysis revealed that while B-cell numbers remained unchanged, there was a significant increase in CD3^+^, CD4^+^, and CD8^+^ T-cells, pro-inflammatory M1 macrophages and neutrophils in TNF-tg^11βKO^ arthritic mice compared to TNF-tg mice.^226^ Collectively, these studies suggest that global endogenous GC signaling, when enhanced by increased 11β-HSD1 activity, has predominately anti-inflammatory effects in the joint (Table 2). Furthermore, to mechanistically determine whether GR dimerization is required for producing the anti-inflammatory effects of GCs in arthritis, GR^dim^ mice have been utilized in both AIA and K/BxN STA mouse models.^214,231^ It has been demonstrated, however, that abrogation of GR dimerization does not alter joint inflammation when compared to wild-type mice, suggesting the anti-inflammatory effects of endogenous GCs may not be mediated through the GR dimerization pathway, at least in AIA and K/BxN STA.^214,231^ Although, these studies did find that GR dimerization was essential in mediating the anti-inflammatory effects of exogenous (therapeutic) GCs.^214,231^

**Table 2:** Effects of endogenous glucocorticoids in genetically modified arthritis mouse models

Cell type	Genetic model	Arthritis model	Key findings	Endogenous GC signaling effect	Ref.
RHEUMATOID ARTHRITIS					
All cells	11β-HSD1 knockout	K/BxN STA	Increased inflammation, joint swelling, exostosis, and ganglion cysts	Predominately anti-inflammatory	Coutinho et al.^230^
All cells	11β-HSD2 knockout	K/BxN STA	No difference	11β-HSD2 plays no role in acute inflammation	Coutinho et al.^230^
All cells	11β-HSD1 knockout	TNF-tg	Increased inflammation, bone loss, type 1 macrophages, and pannus invasion.	Predominately anti-inflammatory	Hardy et al.^226^
T, B, myeloid and dendritic cells	GR knockout	AIA	No difference	Cell specific GR knockouts did not alter inflammation	Baschant et al.^214^
Macrophages	11β-HSD1 knockout	K/BxN STA	Impaired resolution, bone erosion, synovial hyperplasia, and increased neovascularisation	Anti-inflammatory	Zhang et al.^235^
Mast cells	1β-HSD1 knockout	K/BxN STA	Increased mast cell degranulation and activation in vitro	Anti-inflammatory	Coutinho et al.^236^
FLS, myocytes, and osteoblasts	11β-HSD1 knockout	TNF-tg	Modest protection from arthritis	Collectively pro-inflammatory	Hardy et al.^226^
Osteoblasts and osteocytes	11β-HSD2-tg	K/BxN STA	Less inflammation, bone, and cartilage damage	Pro-inflammatory	Buttgereit et al.^240^
Osteoblasts and osteocytes	11β-HSD2-tg	CAIA	Less inflammation, bone, and cartilage damage	Pro-inflammatory	Tu et al.^241^
Osteoblasts and osteocytes	11β-HSD2-tg	AIA	No difference	Bone cells do not modulate T-cell-mediated immune responses via GCs	Spies et al.^242^
Chondrocytes	GR knockout	AIA, K/BxN STA	Increased joint swelling and inflammation (in both models)	Anti-inflammatory	Tu et al.^238^
OSTEOARTHRITIS					
Osteoblasts and osteocytes	11β-HSD2-tg	DMM	Decreased cartilage degradation, osteophyte size, and subchondral bone sclerosis	Destructive	Tu et al.^252^
Chondrocytes	GR knockout	DMM	Decreased cartilage degradation	Destructive	Macfarlane et al.^264^

*GC* glucocorticoid, *GR* glucocorticoid receptor, *11β-HSD1 and 2* 11β-hydroxysteroid dehydrogenase enzymes 1 and 2, *FLS* fibroblast-like synoviocytes, *K/BxN STA* K/BxN serum-transfer arthritis, *TNF* tumor necrosis factor, *Tg* transgenic, *AIA* antigen-induced arthritis, *CAIA* collagen-antibody-induced arthritis, *DMM* destabilization of the medial meniscus

#### The effects of endogenous glucocorticoid signaling in arthritis are cell specific

##### Immune cells

To study the effects of endogenous GC signaling in individual immune cells, a number of cell-targeted conditional GR knockout mouse lines have been established by crossing GR^flox/flox^ mice^232^ with various Cre mice and used in conjunction with arthritis models. Antigen-induced arthritis (AIA) is a T-cell-dependent arthritis model where inflammation is mediated by dendritic cells, B-cells, neutrophils, and macrophages, making it the most appropriate RA model for studying the actions of GCs in these cell types. Surprisingly, conditional deletion of the GR in either T-cells (GR^LckCre^ mice), B-cells (GR^CD19Cre^ mice), myeloid cells (GR^LysMCre^ mice), or dendritic cells (GR^CD11cCre^ mice), did not alter inflammatory progression of AIA in mice when compared to their GR^flox^ control.^214^ It was however found that the GR in T-cells specifically mediated the anti-inflammatory effects of dexamethasone given to GR^LckCre^ mice.^214^ In addition, dexamethasone treatment in IL-17A knockout mice was unable to reduce clinical signs of AIA, implying that IL-17-producing T-cells are the most important targets for the anti-inflammatory effects of GC treatment. The finding that arthritis was affected in GR^LckCre^ mice only when dexamethasone was administered is possibly because the actions of GCs are dose dependent and dexamethasone has much higher potency than cortisol. For example, in rats, removal of endogenous GCs by adrenalectomy or treatment with low-dose corticosterone results in increased macrophage nitric oxide and cytokine (TNF-α, IL-1β, IL-6, and IL-12) production, suggesting basal amounts of GCs enhance immune function in the event of adverse stimuli.^233^ In contrast, treatment with high-dose corticosterone in rats was found to repress macrophage nitric oxide and cytokine production, preventing an exaggerated immune response.^233^ Thus, it is important to appreciate that the actions of endogenous and exogenous GCs are inherently different based on their respective concentrations and pharmacological potency.

In mice with conditional deletion of the GR in macrophages, stimulation of toll-like receptor 4 by lipopolysaccharide results in increased mortality and pronounced cytokine production of TNF-α, IL-1β, IL-6, and IL-10^213,234^ suggesting GR signaling in macrophages has anti-inflammatory effects in sepsis and inflammatory diseases. More recently, targeted 11β-HSD1 knockout in macrophages was found to impair resolution of K/BxN STA in mice, to a similar extent seen in 11β-HSD1 global knockout arthritic mice.^235^ The arthritic joints of 11β-HSD1 macrophage-knockout mice displayed significantly higher bone erosion and synovial hyperplasia compared to control mice. In addition, 11β-HSD1 macrophage-knockout arthritic mice had greater neovascularization of their joint tissue, suggesting that GC signaling in resident macrophages plays an important role in the resolution of inflammation.^235^ In addition, Coutinho et al.^236^ found that mice with targeted 11β-HSD1 knockout in mast cells have an altered mast cell phenotype characterized by a higher proportion of lighter granules and empty vesicles, compared to control mice. This feature is typical of piecemeal degranulation which is associated with chronic inflammation. Furthermore, stimulation of these 11β-HSD1 knockout mast cells with K/BxN serum in vitro reduces the activation threshold of the cells, resulting in extensive degranulation compared to control. However, the functionality of 11β-HSD1 mast cell-knockout in vivo is still unknown.^236^ Nevertheless, it seems that macrophages and mast cells are central targets of GC signaling in mediating anti-inflammatory pathways in arthritis (Table 2).

##### Chondrocytes

Although chondrocytes are one of the dominant cell types in the joint, research into their role in RA has been somewhat neglected. This is partly because chondrocytes have been viewed as passive cell targets of the pro-inflammatory environment, which causes their apoptosis and degradation of the extracellular cartilage matrix.^237^ As a consequence, little is known whether chondrocytes contribute to the severity of the inflammatory process in arthritis and whether these cells are directly targeted by endogenous GCs. To investigate the contribution of chondrocytes to arthritis, Tu et al.^238^ used a tamoxifen-inducible chondrocyte-specific GR knockout (Col2a1-CreER^T2^; GR^flox/flox^) mouse line. Abrogation of chondrocyte GC signaling in K/BxN STA resulted in increased joint inflammation in both the acute and subacute inflammatory phases. This coincided with an upregulation of pro-inflammatory intra-articular cytokines such as IL-1, neutrophil-recruiting chemokines, CXL2/5 and their receptors CXCR2 in the acute phase, and MMPs in the subacute phase. Most interestingly, CXCR2^+^ LY6^+^ CD11b^+^ splenic neutrophils were significantly increased in chondrocyte-GR knockout K/BxN STA mice suggesting that under control of GCs, chondrocytes communicate with leukocytes for neutrophil recruitment through CXL2/5–CXCR2 chemokine axis.^238^ In an AIA model, exaggerated joint inflammation in chondrocyte-GR knockout mice was also observed, indicating that through endogenous GC signaling, chondrocytes seem to not only regulate innate immune responses, but also adaptive immune pathways.^238^ It therefore appears that endogenous GC signaling in chondrocytes has important anti-inflammatory actions, along with macrophages and mast cells.

##### Osteoblasts and osteocytes

Since bone erosion is a hallmark feature of RA, the extent to which the cellular metabolism of endogenous GCs in osteoblasts and osteocytes modulates RA is of particular interest. Persistent inflammation and elevated intra-articular levels of TNF-α and IL-1β stimulate local osteoclast formation and progressive bone erosion in RA. It is well established that these cytokines also affect the osteoblast by increasing RANKL expression and inhibiting bone formation at disease relevant sites, further contributing to arthritic bone loss.^80–82^ However, little is known about the effects of these pro-inflammatory cytokines on intracellular GC levels in bone and how this modulates the arthritic and destructive RA process. As GC therapy is known to induce bone loss through osteoblasts and osteocytes specifically, these cells are of particular interest in this regard. Thus, a transgenic mouse line in which 11β-HSD2 is expressed using the 2.3 kb collagen type Ia1 promoter (Col2.3-11β-HSD2-tg mice) has been developed. In this model, the 11β-HSD2 transgene is expressed exclusively in mature osteoblasts and osteocytes. The ectopic overexpression of 11β-HSD2 effectively abrogates GC signaling in these cells and as a consequence, Col2.3-11β-HSD2-tg mice are largely protected from GC-induced bone loss.^239^

Surprisingly, when K/BxN STA was induced in Col2.3-11β-HSD2-tg mice, abrogation of GC signaling in osteoblasts significantly attenuated joint inflammatory activity and cartilage damage compared to arthritic wild-type littermates.^240^ Clinical and histological analyses revealed that joint inflammation in Col2.3-11β-HSD2-tg K/BxN STA mice was attenuated at the post-acute phase of inflammatory arthritis (day 7 onward), but not in the acute phase (day 0–6), suggesting that the acute stage of K/BxN STA may be regulated by different mechanisms independent of GC signaling in osteoblasts.^240^ The observation that disruption of endogenous GC signaling in osteoblasts and osteocytes protects against joint inflammation and damage in K/BxN STA was confirmed in an alternative arthritis model, namely CAIA. In the CAIA model, different antigen specificity leads to distinct localization of disease, generating much more widespread arthritis involving the paws, elbows and knee joints. While wild-type CAIA mice developed extensive arthritis, Col2.3-11β-HSD2-tg mice with CAIA again displayed a significant reduction in inflammation and protection against bone and cartilage destruction in all joints.^241^ Collectively, the data from these two T-cell-independent arthritis models indicate that osteoblasts and osteocytes, under the control of endogenous GCs, are critically involved in the maintenance of the local joint inflammatory response. In addition, these studies show that GC signaling in these cells can affect inflammation-induced cartilage and bone damage once the acute immune-mediated arthritis has been established. This then raises the question whether GC signaling in osteoblasts and osteocytes is involved in the autoimmune inflammatory response. In an AIA model, which incorporates the adaptive immune response, Col2.3-11β-HSD2-tg mice displayed similar knee joint inflammation to that seen in their arthritic wild-type littermates.^242^ Taken together, these findings indicate that GC signaling in osteoblasts and osteocytes does not affect adaptive T-cell-mediated immune responses, but rather has predominate effects mediating innate immune cell reactions in K/BxN and CAIA models.

It should be noted that clinically, chronic inflammatory processes can lead to systemic bone loss and fractures. Thus, chronic inflammation is a cause of osteoporosis in its own right. Both K/BxN STA and CAIA wild-type mice replicate this feature, displaying significant systemic bone loss associated with increased osteoclast surface and numbers, as well as decreased osteoblast surface, suggesting that inflammation-induced bone loss results from both increased bone resorption and decreased bone formation. In contrast, inflammation-induced bone loss is preventable in Col2.3-11β-HSD2-tg arthritic mice.^240^ These mice have unchanged bone volume and bone turnover, confirming that systemic bone loss is sequelae of active inflammation.

##### Mesenchymal-derived cells

Synovial hyperplasia, also known as the “pannus”, is characterized by the extensive proliferation of mesenchymal-derived FLS and the infiltration of innate and adaptive inflammatory cells.^42–48^ FLS are key mediators of pannus formation and the continued upregulation of pro-inflammatory cytokines. As reviewed above, synovial GC production, through 11β-HSD1 activity is increased by local inflammation and it is therefore possible that GC signaling in FLS plays a role in RA progression. However to date, there is no established model that specifically targets GC signaling in FLS. Instead, to elucidate the role of FLS, models that disrupt signaling in the mesenchymal cell lineage that gives rise to fibroblasts, osteoblasts and chondrocytes have been utilized.

Deletion of the GR in mesenchymal cells leads to perinatal lethality in GR^Dermo1^ mice due to the GR’s essential function in lung development.^197^ To investigate the effects of the GR in arthritic FLS, GR^Col1a2-CreERT^ mice were generated by crossing GR^flox/flox^ mice^232^ with Col1a2-CreER^T2^ transgenic mice, in which Cre is driven by a tamoxifen-inducible, fibroblast-specific collagen type I a2 promoter. This enables inducible and targeted gene deletion in fibroblastic cells at sites of the skin, blood vessel wall, synovial tissue and bone.^243^ While GR mRNA and protein expression was reduced in FLS of K/BxN STA mice, joint inflammation, assessed by ankle thickness and clinical score, was comparable between GR^Col1a2-CreERT^ and GR^flox/flox^ control mice.^231^ Similar results have been found in mesenchymal 11β-HSD1 knockout mice, generated by crossing 11β-HSD1^flox/flox^ mice with Twist2-Cre transgenic mice, in which GC signaling in mesenchymal-derived cell lineages such as FLS, chondrocytes and osteoblasts is disrupted. Using TNF-tg^11βflx/tw2cre^ mice, it was found that disrupted 11β-HSD1 expression in mesenchymal cells resulted in a modest protection from local joint inflammation and destruction, suggesting that these cell types were not responsible for the inflammatory phenotype seen in global 11β-HSD1 knockout mice.^226^ However, as other studies have found GC signaling in mesenchymal-derived cells such as chondrocytes has anti-inflammatory effects and signaling in osteoblasts and osteocytes has pro-inflammatory actions, this may be why the net contribution of mesenchymal-derived cells to RA appears minimal in this paper. Also, as the 11β-HSD1 knockout was across the mesenchymal cell lineage in these TNF-tg^11βflx/tw2cre^ mice, the role of joint FLS cannot be specfically identified.

**Fig. 4:**
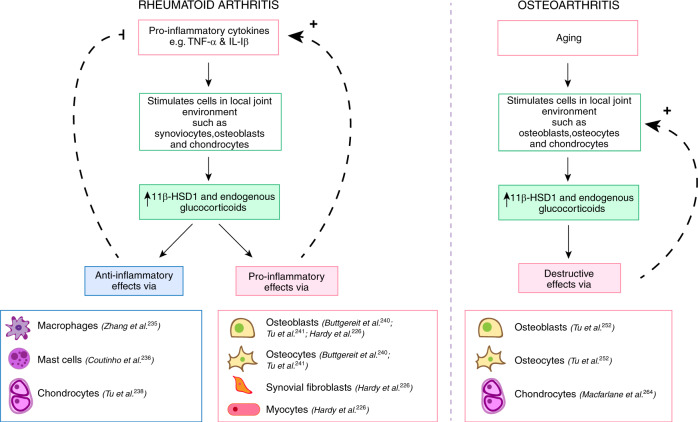
Endogenous glucocorticoid signaling in rheumatoid arthritis and osteoarthritis. In rheumatoid arthritis, pro-inflammatory cytokines increase 11β-hydroxysteroid dehydrogenase type 1 (11β-HSD1) synthesis in various cells of the joint compartment. In osteoarthritis, aging is associated with increased systemic and local glucocorticoid concentrations and 11β-HSD1 expression. 11β-HSD1 is a cytoplasmic enzyme that converts inactive glucocorticoids to their active form, thereby increasing intracellular glucocorticoid concentrations and activation of glucocorticoid-dependent signaling pathways. Depending on the stimulated cell type, this may result in anti-inflammatory or pro-inflammatory glucocorticoid signaling cascade that either suppresses or perpetuates the feedback loop in the local environment

Together these studies, which have utilized various genetically modified mouse models that disrupt or inhibit GC signaling, have enhanced our understanding of the role of endogenous GCs in RA. Current evidence indicates that the pro-inflammatory arthritic microenvironment enhances 11β-HSD1 expression and local GC availability and activity. While this increase in endogenous GCs appears to have predominately anti-inflammatory effects, as demonstrated by global 11β-HSD1 and GR inhibition, endogenous GCs may also play a role in the progression of RA, depending on the cell type involved. Endogenous GC signaling in certain cells such as macrophages, mast cells and chondrocytes seem to produce anti-inflammatory effects whereas signaling in osteoblasts, osteocytes, synovial fibroblasts, and myocytes seem to result in pro-inflammatory and immunostimulatory actions (Fig. 4, Table 2).

### Endogenous glucocorticoid signaling in osteoarthritis

#### Endogenous glucocorticoid signaling increases with age

Evidence indicates with aging there is an increase in systemic and local cortisol levels that together have detrimental effects on normal stress responses, bone health, as well as OA susceptibility and progression. Under normal physiological conditions, adrenal cortisol secretions display a diurnal rhythm with peaks in the early morning and mid-afternoon. With advancing age this pattern becomes more irregular, characterized by a flattening of the cortisol diurnal profile.^244^ Age-related increases in GCs may be caused by a decreased systemic response to negative feedback at the level of the HPA axis due to reduced hypothalamic GR expression^245^ and/or an increase in 11β-HSD1 activity locally in specific tissues.^246,247^ Increased systemic GC levels but in particular, enhanced local availability of endogenous GCs during aging, may lead to reduced bone mineral density and an increased risk of fracture. Both high levels of exogenous or endogenous GCs suppress bone formation and promote osteoblast and osteocyte apoptosis, causing an imbalance in bone remodeling.^85^ Studying aged mice, Weinstein et al.^247^ found that increased adrenal production of GCs and skeletal expression of 11β-HSD1 was associated with decreased bone vascularization. Furthermore, when GC signaling was inhibited in bone by transgenic expression of 11β‐HSD2 (OG2-11β‐HSD2-tg), mice were protected from age-related adverse effects including loss of bone mass and strength.^247^

In OA, which predominately occurs in the elderly, an age-related increase in skeletal GC signaling may negatively impact disease susceptibly and pathology. To date, there are very few clinical studies that have investigated associations between OA and endogenous cortisol levels or the HPA axis in patients. To our knowledge, only one study has investigated whether the HPA axis is dysregulated in OA patients.^248^ The authors determined plasma and urinary cortisol levels over 24 h (along with a range of other hormones) in 35–65-year-old males with or without OA. Interestingly, ACTH peak area and height were slightly lower in OA patients compared to age-matched healthy controls. However, as this difference did not affect overall ATCH or cortisol serum concentrations, the physiological significance of the findings remain uncertain.^248^ While OA itself may not be associated with abnormal changes in the HPA axis or circulating GC levels when compared to *age-matched* healthy controls, this study did not take into account that endogenous GCs increase with age. As such, cortisol levels in a 35-year-old OA patient may be inherently different to a 65-year-old patient. Therefore, when interpreting these results, it is important to consider that OA is an aging disease, and as systemic and local GC levels increase with age, this may contribute to OA pathology regardless of whether the HPA axis is abnormal.

#### Systemic cortisol levels and pain

As intra-articular cortisol injections have been used to reduce pain in OA patients, other studies have focused on whether endogenous cortisol levels are associated with pain. Results from two studies suggest possible gender related differences in cortisol and pain associations, which has been reported in other non-OA related stress studies.^249^ Carlesso et al.^250^ found that pain was positively associated with increased saliva cortisol levels throughout the day in female OA patients. In this study however, cortisol levels upon awakening were not measured and this would have provided a better indication whether the diurnal pattern of cortisol secretion was also disrupted in these OA patients.^250^ On the other hand, Khoromi et al.^248^ reported no differences in plasma and urinary cortisol levels between male, age-matched OA patients with chronic pain and healthy controls. Ethnic differences have also been reported by Herbert et al.^251^ who found plasma cortisol levels were negatively associated with pain in non-Hispanic White, but not African American OA patients during a cold-pressor task. While together these studies suggest gender and ethnicity are important co-factors in interpreting the role of endogenous GC signaling in OA, these studies have limitations in that each used different cortisol collection methods and the majority did not investigate diurnal rhythms of cortisol secretion or variations in the HPA axis. To validate the effects of gender and ethnicity on pain and cortisol levels it is important to collect cortisol levels at the same time intervals between groups over 24 h, given the diurnal rhythm of cortisol secretion.

#### Disruption of endogenous glucocorticoid signaling in bone cells attenuates osteoarthritis

The study of genetically modified mice in which endogenous GC signaling has been disrupted in specific target cells and tissues has provided valuable insight into how endogenous GCs may be involved in the pathogenesis of OA. A recent study from Tu et al.^252^ found that disruption of GC signaling by 11β-HSD2 overexpression in osteoblasts and osteocytes (Col2.3-11β-HSD2-tg) attenuated the development of OA induced by destabilization of the medial meniscus (DMM-OA) in older but not younger mice (Fig. 4, Table 2). When DMM-OA was induced at 22 weeks of age, Col2.3-11β-HSD2-tg mice had significantly reduced subchondral bone sclerosis, cartilage damage, and osteophyte size compared to age-matched wild-type mice, while no differences were found in 10-week-old mice, independent of whether skeletal GC signaling was intact or disrupted.^252^ This data not only implies that GC signaling in bone cells plays an important role in the pathogenesis of OA, but also highlights that this pathway is related to the aging process itself, as skeletal 11β‐HSD1 mRNA expression increased with age in these mice. Correspondingly, OA pathology was more severe in older mice compared to younger mice,^252^ supporting the hypothesis that increases in local 11β-HSD1 expression may enhance destructive GC signaling pathways in bone cells with age, considering the DMM-OA model induces minimal local inflammation itself. In addition to reductions in subchondral bone sclerosis and osteophyte formation, the older transgenic mice also displayed less cartilage destruction, on first sight a counterintuitive finding.^252^ However, this result further alludes to the hypothesis that changes in bone cells and subchondral bone structure may influence the integrity of the articular cartilage and the functioning of other joint cells such as chondrocytes. Unfortunately, the study by Tu et al.^252^ did not investigate the mechanism of how osteoblasts facilitate pathological aspects of OA, although a potential pathway may be through Wnt/β-catenin signaling.^253^ The Wnt/β-catenin pathway has been well described in OA pathogenesis and is known it play an important role in the disease (reviewed in^254^). In fact, a small molecule Wnt inhibitor (Lorecivivint, SM04690) has provided encouraging results as a disease-modifying OA drug, improving pain, function, and joint space width, in recent phase I and II clinical trials (NCT02095548).^254,255^ Wnt/β-catenin activity is increased in both OA subchondral bone and cartilage^256^ and inhibition of Wnt signaling in menisectomized mice by transgenic expression of Dickkopf-1 (Dkk-1), a Wnt antagonist and known target of GCs,^256,257^ can reduce OA severity.^256^ In addition, evidence suggests that Dkk-1 can reduce cartilage degradation by inhibiting vascular endothelial growth factor (VEGF) expression in bone,^256^ providing a possible “cross-talk” mechanism by which bone directly influences cartilage pathology. Again, this suggests the health of the articular cartilage depends on the stability of the underlying subchondral bone and that these two tissues work synergistically in OA to ultimately support the loading of the joint. Disruption of GC signaling in osteoblasts and osteocytes by 11β‐HSD2 transgenic expression results in reduction of Wnt10b and Wnt7b mRNA and β-catenin protein levels and markedly reduced osteoblastogenesis during development.^258^ Thus, it is possible that endogenous GCs mediate or promote the development of OA through Wnt/β-catenin and Dkk-1 signaling. Another important coordinator of bone remodeling and potential GC target in OA is TGF-β. While TGF-β has been shown to be regulated by GCs in other disease contexts,^259,260^ it remains unclear if this is the case in OA. Nevertheless, these studies are important in highlighting the role of bone remodeling mediators in OA pathogenesis, particularly as the majority of aging-related studies in OA have focused on chondrocyte biology. The precise abnormal mechanisms that occur between the subchondral bone and articular cartilage in OA initiation and progression remain interesting areas of research and so far, suggest GCs, Wnt/β-catenin, Dkk-1 and TGF-β may play key roles.^253^

#### Evidence for the role of endogenous glucocorticoid signaling in chondrocytes

While GC signaling in bone cells appears to have destructive effects in both OA and RA, the contribution of GC signaling in other cell types such as chondrocytes is still under investigation. Interestingly, endogenous GC excess (such as Cushing’s disease) and long-term GC therapy are both associated with defects in chondrocyte function, characterized mostly by growth retardation in adolescents^261,262^ and cartilage degradation in arthritis.^12^ In addition, it is currently understood that exogenous GCs mediate both favorable and adverse effects on cartilage and chondrocytes depending on their concentration and duration of use, which may suggest endogenous GCs could also have effects in the maintenance of OA cartilage.^11,12,190^ DiBattista et al.^263^ demonstrated the GR is downregulated in OA chondrocytes compared to aged-matched normal chondrocytes, however, the physiological significance of this is unclear. More recently, we have observed that targeted GR knockout in chondrocytes of older (22-week-old) mice attenuates cartilage destruction following DMM surgery.^264^ This result indicates that GC signaling in chondrocytes produces predominately destructive effects in OA, but anti-inflammatory effects in RA murine models. Therefore, not only are the effects of endogenous GC signaling cell specific, but their actions also depend on the nature of the pathological condition or process. This then raises concerns over the appropriateness of glucocorticoid therapy in OA.

Interestingly, recent studies investigating circadian chondrocyte biology in OA have highlighted a potential pathway in which endogenous GCs may mediate OA. The diurnal rhythm of cortisol and melatonin secretion are tightly controlled by the suprachiasmatic nucleus in the hypothalamus.^265^ Aging is associated with a disturbance of the body’s autonomous circadian clock as well as the loss of cortisol diurnal patterns. While there is a reduction in melatonin, which can impair sleep quality and increase the risk of neurodegeneration, cortisol levels become elevated with age.^244,265^ Endogenous GCs play an essential role in the regulation of circadian rhythm through interactions with the circadian clock system, a self-driven molecular pacemaker consisting of the CLOCK-BMAL1 transcription factor heterodimer.^266^ GCs and the circadian clock system interact with each other in both the hypothalamus and peripheral tissues where GCs have been shown to induce expression of clock genes and clock proteins can directly bind to the GR and repress its actions.^266,267^ In the context of OA, the clock gene *BMAL1* has been identified as an important regulator of cartilage integrity in chondrocytes.^268,269^ Dudek et al.^268^ found both OA and aged chondrocytes have reduced expression of BMAL1 and chondrocyte-specific *BMAL1* knockout mice, developed OA-like cartilage lesions characterized by chondrocyte apoptosis and matrix damage, that was more severe with increasing age. Similarly, Snelling et al.^269^ demonstrated that knockdown of BMAL1 in human chondrocytes in vitro results in increased cell proliferation and MMP-13 gene expression which is a major cartilage degrading enzyme in OA. Although the interaction with GCs was not examined in these studies, it is possible that dysregulation of BMAL1 may induce abnormal changes in GC signaling in joint tissue, considering endogenous GCs and circadian rhythm are closely associated. Nevertheless, these studies provide interesting insight into the importance of autonomous circadian rhythms in chondrocyte health and maintenance of cartilage integrity that may contribute to arthritis susceptibility if they become dysfunctional.

In summary, endogenous 11β-HSD1 expression in the OA joint increases with age,^252^ possibly due to age-related chronic low-grade inflammation. To date, there are limited studies that have modeled endogenous GC signaling in OA and this warrants further investigation to determine molecular signaling pathways involved in the progression of disease. Interestingly, the disruption of GC signaling in both bone cells *and* chondrocytes can attenuate OA in a DMM mouse model,^252^ suggesting GCs play a damaging role in OA.

## Conclusions

RA and OA, while differing in etiology, both lead to progressive destruction of the joint. While RA is driven by a systemic autoimmune inflammatory reaction, OA is primarily a local degenerative condition where aging as well as biomechanical and metabolic factors interact in a complex and varying pattern. Since the pathomechanisms underlying these two forms of arthritis are different, the effects of pharmacological and endogenous GCs also vary between the diseases. In RA, the pro-inflammatory microenvironment stimulates the expression of 11β-HSD1 and thereby local endogenous GC activity in joint cells. Endogenous GC signaling in macrophages, mast cells, and chondrocytes produces anti-inflammatory effects. Although endogenous GC signaling in osteoblasts and osteocytes appears to promote inflammation, the overall actions of endogenous GCs in RA are predominately beneficial and reduce inflammation. In OA, endogenous 11β-HSD1 expression and local endogenous GC activity are also increased in the joint, mostly due to aging-related mechanisms. The effects of endogenous GC signaling in both osteoblasts and chondrocytes, however, has destructive effects in OA. This suggests that endogenous GCs are predominately detrimental in pathogenesis of OA.
